# Competency in Object Control Skills at an Early Age Benefit Future Movement Application: Longitudinal Data from the NW-CHILD Study

**DOI:** 10.3390/ijerph18041648

**Published:** 2021-02-09

**Authors:** Anita E. Pienaar, Carli Gericke, Wilmarié du Plessis

**Affiliations:** PhASReC, School of Human Movement Sciences, North West University, Potchefstroom 2520, South Africa; gerickecarli@gmail.com (C.G.); 20376138@nwu.ac.za (W.d.P.)

**Keywords:** motor competence, fundamental motor skills, object control skills, early childhood, later childhood, health, active lifestyles

## Abstract

The level of competency in object control skills (OCSs) during early childhood is considered to be a possible determinant of the successful generalization of these skills during later childhood. This study aimed to determine if an association exists between competency in object control skills during early childhood (6–9 years) and the application of these skills during later childhood (12 years). The NW-CHILD longitudinal study (2010–2016), included a baseline and two time-point follow-up measures in grades 1, 4, and 7 of South African children. A total of 374 participants (boys = 178, 47.59% and girls = 196, 52.41%) completed testing at all three time-points and were analyzed. The Test of Gross Motor Development, Second Edition, and the Canadian Agility and Movement Skill Assessment were used to determine associations between object control skill competency during early and later childhood by using descriptive statistics, Spearman rank order correlations, and stepwise regression analysis. The level of object control skill competency at 6 and 9 years, significantly influences the application of these skills at 12 years. A high overall and significant contribution of OCS (4.6%, *p* < 0.01) to the variance in the skills and time scores at 12 years; *p* < 0.05 were found. Competence in object control skills at an early age can provide a baseline from where opportunities for progression or transfer of skills can result in more advanced skillful executions which consequently can be considered to be a cornerstone of improved future physical activity and healthier lifestyles.

## 1. Introduction

Fundamental motor skills (FMSs) are considered to be an important aspect of general motor competence, therefore the promotion of FMS is considered to be integral to a holistic view of development [1,2]. The promotion of fundamental motor skills (FMS) development in young school children is motivated by the scientific evidence related to public health, psychosocial benefits, and positive academic outcomes associated with the acquisition of motor skills [1,2,3,4,5,6]. Associations are drawn between FMS not only laying the foundation for future movement but also for physical activity and adopting physically active lifestyles [1,2,3]; physical fitness (PF) [4], healthy weight status, [5] improved cognitive outcomes [6] and social skills [7]. Object control skills (OCSs), which are considered to be a subcategory of FMS (i.e., throwing, kicking, catching, rolling, dribbling), also referred to as manipulative skills, demand high levels of functional coordination and control to make controlled contact with objects in one’s environment which requires the controlling of objects with the hand, foot or implements [2]. These skills can be regarded as core and baseline skills to provide children with the best possible chance to successfully and continually engage in a range of physical activities and more specifically health enhancing activities [2,8,9].

During early childhood, young children are involved in the process of developing fundamental movement skills in a variety of movements and should be able to transfer these different motor proficiency (MP) capabilities into sport-specific and specialized skill environments during the middle and later childhood years (7 years and older) [8,9]. During the sport-related movement development phase, which is described as the age period between 7 and 10 years, children are exposed to different sport cultures and sport activities in school and at home and are more likely to become more interested in sport participation [9]. Skills should, therefore, be developed during this developmental period into more specialized sport-specific skills that can be applied and transferred to various sports during this period [9]. It is important to note that FMS and especially OCS do not only develop during free play [10] but opportunities for skill-related instruction and repetition are important for children to become skilled movers [9]. Well-developed FMS, and more specifically OCS skills, therefore open up opportunities for adapting or varying the context at different levels across the lifespan [9].

Obtaining competency in FMS cannot be considered in isolation as developmental change in a child’s motor skills is influenced by many critical determinants. Two factors that should always be taken into consideration concerning sport-related competency, are biological and environmental constraints [11]. It is stated [2] that motor development in young children, specifically during the early childhood years, is influenced firstly by biological maturation, and only then the influence of practice and opportunity becomes important.

Biological differences in OCS competency that were evident as sex differences are reported by researchers [12,13,14]. Studies in America [12] Australia [13] and South Africa (SA) [14] confirmed boy-girl differences, favoring OCS competence in boys between the ages of 5 and 16 years. Another sex-related study [15] furthermore suggests that sex may be an important factor in motor performance and especially in motor learning. The researchers report a male advantage in motor learning rather than in motor performance per se, and this advantage was enhanced during adolescence. These researchers also reported that tasks that involve gross body movement, rather than fine movements often show advantages of males over females in terms of both speed and accuracy [15]. Various researchers [1,14,16,17,18] also report that socio-economic influences play a significant role in obtaining movement proficiency. An environment poor in resources is considered to hamper motor development.

Children’s demonstration of competency in motor skills is, therefore, considered to be a cornerstone leading to their future physical and motor skill development as FMSs are commonly used in many forms of sports and physical activities [9,19,20]. The association between FMS and sport-specific outcomes is also highlighted by various researchers [2,7,21]. These researchers are in agreement that successful participation in sport is influenced by basic learned movement patterns that are foundational to more complex physical and sporting activities. In particular, the association between the competency in object control skills (OCSs) and specialized sport-related skills and sports participation have been highlighted in recent studies [2,12,13,14,22,23,24]. It is noted that children with inadequate OCS tend to avoid participation in sport activities because they struggle to master advanced skills [1,2]. Feeling competent will therefore motivate children to participate in a variety of physical activities without the stress of embarrassment and instead with feelings of confidence and competence (i.e., perceived motor competence) [25]. Children who feel competent are also better able to cope with the demand of participating in sporting activities and may therefore be more physically active through sport, gaining additional physical and mental health benefits. Increasing positive self-perceptions of motor skill performance through actual motor competence may therefore be important for facilitating participation in sport and physical activity [1,25].

Researchers [2,5] stated that future research should continue to examine what needs to be done to promote the development of more physically active children and whether fundamental motor skills provide the foundation for lifelong skills and health-enhancing forms of physical activity. Although a strong rationale for the development of FMS is evident from the literature, longitudinal studies are especially recommended to examine the relationship between subcategories of fundamental motor skills (i.e., locomotor or object control skills) and future health outcomes [2]. Most longitudinal studies have studied the association between early OCS and physical activity [7,26,27] or physical fitness [28]. The tracking of these skills is, however, less studied in longitudinal studies. Tracking is an indication that measurements tend to follow a pattern over a period of time in which initial measurements predict later levels. One longitudinal study conducted in New South Wales tracked kicking, catching, and overhand throwing from 10.06 to 16.44 years and established that childhood object control proficiency significantly predicted adolescent object control proficiency [13]. The skills were assessed at each time point of the study based on six features that were considered integral to the proficient performance of the skill, but without taking into account that at older ages, OCS skills mostly have to be applied in more complex environments where proficiency, as realized in accuracy and timing of performance might be influenced by external factors. The possible association between early competency in OCSs and the application of these skills in more dynamic environments at older ages (thus the transfer or tracking likelihoods of these skills during childhood) are, however, not studied and also needs an improved understanding. This study aims to provide more insight into this knowledge gap, by using longitudinal data of OCS of children living in the North West Province of SA. This study examined the assumption that being proficient in OCS during earlier childhood will increase opportunities to refine these skills into more advanced skills and thereby will enhance the likelihood of application and transfer of these skills in more complex environments during later childhood.

## 2. Materials and Methods

### 2.1. Study Design

The study is based on a longitudinal research design and forms part of the North West Child Health, Integrated with Learning and Development longitudinal study (NW-CHILD study) which spans the primary school period of seven years and was conducted from 2010–2016 in South Africa. The research group was selected by means of a stratified random sample in conjunction with the Statistical Consultation Services of the North West University, Potchefstroom, SA. To recruit participants, a list of all the schools in the North West Province of SA was obtained from the Department of Education in the North West Province of SA. Stratification was conducted for school districts, school quintiles (1–5), and sex (male and female). All the schools in this province are grouped into eight educational districts, each consisting of 12 to 22 regions with approximately 20 schools (minimum 12, maximum 47) per region. From four different educational districts, 20 schools, representing five different school quintiles, and children of both sexes, were recruited to take part in the study. The North West Department of Education in SA used a poverty classification in each province to classify schools in different quintiles (Quintile 1, i.e., school types from low socio-economic status (SES) backgrounds, to Quintile 5, that is, school types associated with higher SES backgrounds). The quintile status of South African schools is determined by the National Treasury and according to the National Poverty Table, obtained from the National Census data, including income, dependent ratios, and levels of literacy [29]. Quintile 1 and 2 schools are the poorest schools and are exempted from paying any school fees according to Statistics South Africa in 2010 [29]. The North West Province of SA is characterized by high poverty, especially in rural areas, unequal distribution of income between different population groups, and unemployment (Statistics South Africa, 2010). In 2010 and 2013, data from the Test for Gross Motor Development, Second Edition (TGMD-2) were used to measure fundamental motor skills. In 2016, measurements resulting from the completion of a motor skill course, namely the Canadian Agility and Movement Skill Assessment (CAMSA) was used as a proxy to determine OCS competency during later childhood. The quantitative data obtained from the TGMD-2 (2010 and 2013), and the CAMSA (2016) were integrated into this study to answer the research question.

### 2.2. Participants

The same participants who completed the grade 1 and grade 4 measurements were again assessed in grade 7. In grade 1, 864 children were recruited, and 816 were included in the study after their parents/guardians provided parental permission. This group included 419 boys and 397 girls with a mean age of 6.84 + *SD* years (*SD* = standard deviation). In their grade 4 year, they had a mean age of 9.9 years + 0.42 years, with a mean age of 12.9 + 0.41 years in their grade 7 year. Of these participants, 374 (178 boys (47.59%) and 196 (52.41%) girls took part in all three time-point measurements, 214 (90 boys and 124 girls) were from quintile 1–3 schools representing low socio-economic status (SES) settings, while 160 participants (88 boys and 72 girls) represented higher SES settings, based on their school status (quintile 1 and 2 schools). The loss of subjects over the study period was 442 (54.1%). Migration from schools, lack of parental consent, or absence on the day of testing was the main reasons for this large loss of participants over the seven school years.

### 2.3. Measuring Instruments

#### 2.3.1. Test of Gross Motor Development, Second Edition (TGMD-2)

The TGMD-2 is a norm-referenced measure designed to test the gross motor functioning of children 3 to 10 years old. The test consists of 12 motor skills and is divided into two sub-tests, namely locomotor (run, hop, gallop, leap, horizontal jump, and slide) and OCS (overhand throwing, catching, underhand rolling, dribbling, kicking, and striking a stationary ball) [30]. Only the OCS-subtest was used for this study to determine proficiency in six OCS of children aged 6 and 9 years in their grade 1 (2010) and grade 4 (2013) school years. Each of these skills includes several behavioral components presented as performance criteria and in general presents a mature pattern of the skill. If participants performed an action correctly, they received a score of one; if they performed it incorrectly, they received a zero. Each participant received two attempts to perform each skill after a visual demonstration of each skill was performed by the tester. The scores of the two attempts were added together. Standard scores for the sub-items were calculated from the raw scores and then combined to obtain a gross total motor score from which a motor quotient was calculated. The Gross Motor Quotient is the best measure of an individual’s overall gross motor ability [30]. Age, gender, and a raw score of each child were used to calculate the percentile rank as well as the standard score. A child’s performance in comparison with their chronological age group can be obtained by using percentile scales. The descriptive proficiency ratings for subtest standard scores in the TGMD–2 manual is indicated as very superior (17–20), superior (15–16), above average (13–14), average (8–12), below average (6–7), poor (4–5), and very poor (1–3). The TGMD-2 has a validity of r = 0.89 [30]. This test battery was substituted during the 2016 measurements with the Canadian Agility Movement Skill Assessment (CAMSA) which includes most of the FMSs of the TGMD-2, (specifically throwing, catching, and kicking) but is also considered to be a more appropriate test to use among 12-year-olds as it provides a more accurate representation of the application of OCS in a more dynamic environment. The CAMSA assessment represents a developmentally appropriate setting that simulates a sport-related setting and considers the interaction of the individual and the environment, which makes it possible to draw associations between OCS competency during earlier childhood and application of these skills during later childhood.

#### 2.3.2. Canadian Agility and Movement Skill Assessment (CAMSA)

The Canadian Agility Movement Skill Assessment (CAMSA) was used [31] in our study to assess the participants at the age of 12 years in their grade 7 (2016) year. This protocol forms part of the Canadian Assessment of Physical Literacy (CAPL-2) and assesses the actual level of movement competence by means of movement skills that are considered to be needed for a physically active lifestyle. These movement skills include most of the fundamental motor skills that are assessed in the TGMD-2. A Delphi process and the validity (75%) and reliability (95%) of the assessment protocol among children in grades 4 to 6 had been established [32,33]. A time and skill score are obtained in the CAMSA. The movement course includes performing a range of skills in a sequence that includes jumping with two feet, sliding, catching a ball, throwing a ball at a target, skipping, hopping on one leg, and kicking a ball through cones. Two demonstrations are provided (one performing each skill slowly so that each criterion could be observed clearly and one demonstration completing the whole course against time). Each participant is allowed two practice trials followed by two timed trials that are determined to the nearest 0.01 s. The skill score is based on a criterion-referenced assessment of the different skills where one point is awarded when a performance criterion is met. A range of 0 to 14 can be scored and are allocated as follows: two-foot jumping (two marks), sliding (three marks), catching (one mark, if the participant could catch the ball that is thrown to him), throwing (two marks, if the participant threw with an overarm technique and hit the target), skipping (two marks), one-foot hopping (two marks), kicking (two marks, follow through and kicked through the cones). More details can be found on the CAPL website (Canadian Assessment of Physical Literacy, 2nd edition, 2017). The CAMSA assessment provides a dynamic and authentic environment where the speed and accuracy of a participant’s movement execution can be assessed. These characteristics of the CAMSA protocol that considers the interaction of the individual and the environment makes it possible to draw associations between competency in OCS during earlier childhood and the application of these skills during later childhood. In an open environment setting more complex movements (similar to what is needed during organized play and sport participation activities) are needed and combined and should be executed with speed and accuracy and may, therefore, provide insights into sport-related applications during later childhood [27].

### 2.4. Procedures

The Health Research Ethical Committee of the Faculty of Health Science at the North West University, Potchefstroom, SA, granted permission for the longitudinal study (NWU-00070-09-A1, 2009–2015). Parents/legal guardians consented and re-consented via parental permission in 2010, 2013, and 2016. The parents/legal guardians’ permission form thoroughly explains what is to be expected from each participant as well as the risks involved. On the day of field testing, the participants also had to provide written consent after the procedure was explained to them by the principal investigator, and they had time to ask questions. Participants from a total of 20 schools divided into four school districts (five schools per district) were measured during the morning hours of a school day. One school district was assessed over one week (one day of field testing at five different schools) during the summer times of the school calendar (two weeks in April and two weeks in September). The same weeks were used during the follow-up measurements for each school district to ensure more or less the same testing conditions at each follow-up. The participants were under no obligation to participate in the research and could withdraw from the research at any time during the testing.

### 2.5. Statistical Analysis

Analyses were performed using Statistica for Windows (TIBCO Software: Palo Alto, CA, USA) [34]. Data were, firstly, analyzed descriptively (percentages, frequency distributions, means, and standard deviations (*SD*) to describe differences between OCS proficiency groups. The Spearman Rank Order Correlation was used to determine the association between OCS during earlier (6 and 9 years) and later (12 years) childhood and to determine the association of sex, SES, and the object control skill standard score (OCS SS) to the time and total score obtained in the CAMSA. The effect size cut-offs by Cohen [35] were used to determine practical significance. An r ≈ 0.1 was interpreted as a small effect, r ≈ 0.3 as a medium/moderate effect, r ≥ 0.5 as a large effect size. A stepwise regression analysis was then used to determine the association between OCS proficiency during earlier and later childhood. Sex and SES were adjusted for, as possible co-variants in this analysis, where these variables were dummy-coded with boys and low SES were awarded the value of one and girls and high SES the value of zero.

The strength of the association between OCS proficiency during earlier and later childhood controlling for sex and SES in the stepwise regression analysis is presented by the percentage variance explained where R^2^ ≈ 1% can be interpreted as a small effect, R^2^ ≈ 10% as a medium effect, R^2^ ≥ 25% as a large effect. For statistical significance, *p* is set at ≤ 0.05.

## 3. Results

Table 1 provides an overview of the demographic characteristics of the participants of the study. The total number of participants was 374 children of which boys represented 47.59% (*n* = 178) and girls 52.41% (*n* = 196). Regarding SES, 50.57% (*n* = 90) of the boys, and 63.27% (*n* = 124) of the girls fell in the low SES group. The remainder of the boys (49.44% (*n* = 88) and girls (36.73% (*n* = 72)) represented the higher SES group.

Table 2 presents the descriptive statistics of the TGMD-2 OCS SS (object control skills standard score) in the group and per sex in grade 1 and grade 4, and also the skills and timed scores of the CAMSA that were achieved in grade 7. The gender-specific means in the OCS SS increased for boys from grade 1 (7.15) to grade 4 (8.67) as well as in girls in grade 1 (7.43) to 9.62 in grade 4. In grade 7 boys achieved a statistical and practical significantly higher CAMSA skill score (11.69) and a time score (16.51 s) than girls (total skill score: 10.06 and time score: 17.36 s).

Table 3 provides an overview of the possible influences of SES and sex on OCS competency in grades 1 and 4 and the CAMSA skill and time scores that were obtained in grade 7. Statistical significant correlations were established between the OCS SS in grades 1 and 4 and the CAMSA skills and time scores in grade 7. SES in grade 1 showed a small overall correlation (−0.18) with the time obtained in the CAMSA skills score in grade 7. This correlation between grade 1 OCS SS and the CAMSA skills and time scores was of moderate significance for boys (r = −0.39 and r = −0.29) and of small significance for girls (r = −0.23 and −0.18). High SES in grade 1 correlated statistically with both the skills (r = 0.31) and the time score (r = −0.27) while the correlations with low SES were not significant (r = 0.13 and r = −0.11). In grade 4, no correlation between high (r = 0.04 and r = −0.16) and low (r = −0.18 and r = 0.05) SES was found among girls. A higher relationship between sex and OCS proficiency, as opposed to SES and OCS, was also noticed during later childhood. Associations between OCS SS were also higher between the skills score compared to the time scores of the CAMSA.

The results of two different stepwise regression analyses are reported in Table 4 and Table 5.

Table 4 describes the results obtained by stepwise regression analysis regarding the possible association between the OCS skills standard scores that were obtained in grades 1 and 4 with the skills CAMSA score in grade 7. The results showed that 32.6% of the variance could be explained by the results in grade 1, where sex contributed to 16.6%, (*p* < 0.01); SES to 11.4% (*p* < 0.01) and OCS to 4.6% (*p* < 0.01) of the variance. The percentage contribution to the variance of OCS competency in grade 1 and 4 (representations of earlier childhood, as determined by the TGMD-2 OCS SS) is depicted, as well as proficiency and applying of OCS in grade 7 (which represent later childhood, and as determined by the skills and timed scores of CAMSA.

These contributions to the variance can be considered to be of large practical significance as an R^2^ ≥ 25% point to a large effect. In grade 4, 28.1% of the variance obtained in the skills CAMSA score can be accounted for by regression by sex (16.6%: *p* < 0.01) and SES (11.4%: *p* < 0.01), while the OCS SS did not make any contribution to the variance. These results confirmed that proficiency in OCS in grade 1 showed a higher contribution to the variance obtained in the qualitative performance of the CAMSA skills score compared to at age 9, as OCS SS did not enter as a step in the regression analysis (Table 4). The overall contributions of the variables to the variance obtained in the skills score at age 9 (R^2^ = 0.281) can, however, be deemed to be still of large practical significance as an R^2^ ≥ 25% is considered as a large effect.

Table 5 displays the association between the time to complete the CAMSA and the OCS SS that were obtained at ages 6 and 9 years. The results showed that 8.1% of the variance at age 6 could be explained by the steps that entered the regression compared to 4.3% at the age of 9 years. The results also show that OCS competence at age 6 explains a higher percentage of the variance (4.5%) of the time to complete the CAMSA compared to at age 9 (0.9%). The contribution of sex to the variances at ages 6 and 9 years was also very similar, explaining an additional 3.5% and 3.0% of the variance. SES did not enter the stepwise regression at age 6, while it contributed 0.4% of the variance at the age of 9 years.

Competence in OCS at 6 years also showed a bigger contribution to the explained variation in the timed score (time to complete the CAPL) compared to at age 9. The percentage contribution to the CAMSA timed score was, however, much smaller compared to in the CAMSA skills score. In addition, the contribution to the overall variance of the CAPL timed score that could be explained by the steps that entered the regression was also only of small practical significance (since R^2^ = 10% is considered as a medium effect).

## 4. Discussion

This study tested the assumption that being proficient in OCS at an early age will transfer to older ages and will enhance the successful application of these skills in more dynamic sport-related environments during later childhood. This assumption was assessed by means of the CAMSA, where OCSs have to be applied within a motor course that has to be completed against time. When OCSs are mastered a child will be able to execute them with good technique and timing that again will influence the speed and accuracy of execution of these skills [2,9,15].

Our first finding was that competency in OCS skills at a young age (6 years) showed a higher association with the successful application of these skills, compared to at age 9, at older ages in more complex environments. This result might confirm that OCS does not only develop as a result of maturation, but that providing opportunities through structured activities to teach OCS, and informal activities to embrace learned activities, is also important and should be prioritized and formalized within the school environment [2,9,13,14]. It is argued in this regard that when children already obtained good OCS in grade 1, it will provide them with confidence and motivate them to engage in a variety of physical activities, including sport activities during early childhood that can foster and refine the development of these skills even further. It is believed that increasing positive self-perceptions of motor skill performance in children may be important for facilitating participation in sport and physical activity [1]. It is argued in this regard that feeling competent will motivate children to participate in a variety of physical activities without the stress of embarrassment and instead with feelings of confidence and competence (i.e., perceived motor competence) [23,36]. In addition, well-developed fundamental motor skills, especially OCS during the early years, also provide better possibilities for children to be included in sport teams when they are young, where these opportunities will subsequently aid them to advance the further development of OCS in sport-specific contexts. In South Africa, where this study was conducted, school sports include different sport codes that require good ball skills such as rugby, netball, soccer, tennis and cricket [37]. A study confirmed in this regard that children with a higher motor competence choose to participate in a more varied range of physical activities, giving them the opportunity for a variety of movement experiences which might, in turn, result in greater amounts and intensity of physical activity [23]. The significant and consistent contribution of SES influences to the explained variance in grade 1 and grade 4, also confirmed that environmental opportunities play an important role in obtaining full competency during the application of OCS [14,38].

The stepwise regression revealed that the variables that entered into the stepwise regression and influenced the CAMSA time score at the age of 12 years, were in a way different from what was found regarding the variables influencing the skills score. In this analysis, a lower percentage of the variation could be explained by the OCS SS, at both ages 6 and 9. The contribution to the variance (4.6% and 4.5%) in the time and skills scores was, however, similar at the age of 6. At age 9, OCS entered as the first of two steps while at age 6, OCS entered as the third step. In addition, competency in OCS did not contribute to the variance that was found in the skills score at the age of 9, while it showed a contribution of 0.9% to the time score at age 12. This result may be suggestive of more fine-tuned and accurately performed OCS at the age of 9, which was needed to complete the CAMSA sequence as quickly as possible. It was required from the participant in the movement sequence to catch a ball on the run, throw a ball to hit a target, and kick a ball through goal posts accurately, all against time. This finding suggests that OCS needs further fine-tuning and refinement after the age of 6 and the period between 6 and 9 years (after the OCS was mastered), which is rich in opportunities to engage in activities to improve the timing, accuracy, and control requirements of these skills in sport-related environments. Therefore, it can be argued that children who have further improved their OCS during the period of 6 and 9 years were better able to perform these skills under a time constraint. The speed-accuracy trade-off principle thus provides a possible explanation for the difference in the steps that entered at 6 and 9 years to explain the time and total scores at 12. According to Howley [39], it is possible that when a child has to perform skills against time, more errors can occur in the execution of a skill when it is not mastered well. Possible reasons for this may be that the expected time that proficient performances are reached differ between skills. However, between the ages of 5 and 9 years, children are expected to become competent in specific performance criteria for certain skills to enable them to adopt such skills to obtain specific timing requirements [40]. Less skill proficiency can also lead to less control and coordination that can contribute to the child dropping a ball during catching or making a less accurate kick or throw at a target. Also, a child might have well-mastered OCS but might not have been as well-coordinated, influencing the time of completion of the motor course. It should be noted that the movement course also included the execution of locomotor skills, such as, gliding and jumping that requires body coordination [41]. From these results, it seems that once OCS is mastered, it will still be important for children to have opportunities to apply these skills in open-ended environments, as these are the contexts in which they will be used at older ages.

Our results revealed that sex also contributed significantly to the explained variance and played a role in OCS competence. Boys showed a superior skills score (11.69 compared to 10.06) and a time score (16.51 s, and 17.36 s) compared to girls at age 12. The contribution of sex to the variance in the skills score stayed high in grades 1 and 4 (16.6% in both). Regarding the timing score, sex also made a similar contribution in grades 1 and 4 (both 3.0%), although much smaller. This superior performance of boys is also confirmed by other studies [12,13,14,15]. The high value that is placed on boys’ sport participation in SA, such as rugby, cricket, and soccer could have contributed to these sex differences [42]. In both skill scores at 6 and 9 as well as the time scores at 6 and 9, sex was the biggest contributor to the variance; although the contribution was much lower in both the time scores (3.5% and 3.0%). In the timing score sex still contributed to the variance at age 6 and 9 with similar percentages (3.0% and 3.4%) although the percentage was lower compared to the skills score. Here, OCS competency had a higher contribution at the age of six (4.5%) compared to sex (3.5%) while at age 9, sex entered first in the regression (3%) with OCS (0.9%), and SES showing a much smaller contribution (0.4%). It is reported that boys are more proficient in motor skills than girls at all ages and that these differences increase with age, also with regard to speed and accuracy of performance [11,15]. Researchers also reported that boys perform better in speed, agility, reaction speed, strength, and upper-limb coordination tests, while girls show better performance in fine motor skills [15,43]. It is therefore recommended that the development of OCS of both sexes should be considered equally important during early development as OCS can provide a key entrance to sport participation. Researchers [23] believe that children who possess a higher level of motor competency are better able to cope with the demand of participating in sporting activities and may therefore be more physically active through sport, gaining additional physical and mental health benefits.

The variation explained by SES was only evident in the quality of skill execution as evident in the skills score (Table 3 and Table 5) at both 6 and 9 years, where it entered as a second step at 6 years showing a significant contribution to the variance (11.4%). When time was scored, it did not contribute significantly to timing differences at the age of 6, only as a third step, and on a non-significant level at the age of 9 (Table 3 and Table 5). Research also showed that fundamental motor skills proficiency generally increases with increasing socio-economic conditions [13,17,42,44,45]. A South African study among 9-year-olds, confirmed that full mastery of OCS, specifically catching, throwing, rolling, and dribbling, generally increased with higher SES [14]. The results confirmed that the earlier competence in OCS can be obtained, the bigger the transfer effect will be to the application in more complex environments or contexts. On a practical level, the results show that such early competence will provide children with more opportunities and time to apply and further improve or fine-tune these skills during the middle childhood years which will open up opportunities for them to continually and successfully engage in and broadly apply these skills in various activity contexts over the lifespan. It should, however, be noted that although OCS is an important component of fundamental motor skills, competency in OCS cannot alone guarantee positive sport-outcomes or active participation in physical activities. Movement as such is a complex, integrated, synchronized, and multi-jointed activity, inclusive of neuromotor and biomechanical mechanisms that work together, which portrays a complexity of many factors that can influence the development of MP. OCS can, however, provide a firm baseline from where opportunities for progression or transfer of skills can result in more advanced skillful executions. Sex and to a smaller degree, SES, made contributions to the successful application of OCS during later childhood, and these influences should also be addressed to improve early competence in OCS. These results demand action. A national report on the state of Physical Education in South African schools [46] revealed a low status, especially in lower quintile schools as parents, school management and educators emphasize the importance of academic subjects. A lack of access to basic physical resources was also found in these schools. On the other hand, schools in higher SES quintiles predominantly prioritize sporting success and view the importance of Physical Education as a training ground for sporting talent, with the implication that less skilled children may not receive opportunities to improve their basic motor skills. A lack of recognition and resource provision from the Department of Basic Education and the government is also reported in the national report while it is also highlighted that girls are relatively disadvantaged due to the choice of physical activities offered to them. School sports that are offered to young girls that develop object control skills, mainly include netball, compared to boys who have exposure to a bigger variety of object control skills-related sports (rugby, cricket, soccer). A strong health-focused message to school authorities and the government of South Africa is consequently encouraged, which will inform them of the synergistic relationship between sufficient motor competence and physical activity and sport engagement, which may increase positive and healthy behaviors. They should also be informed about the importance of trained, knowledgeable educators with good didactical skills that can deliver developmentally appropriate motor development and physical activity programs that also include a strong mass participation and sport-for-all emphasis in young children. Further studies are also recommended to improve our understanding of the variances that could not be explained by analyzing the role of OCS, sex, and SES in this regard.

Although this study had strengths such as the longitudinal nature of the study that enabled an in-depth look into the association of obtaining OCS proficiency at a young age and the application of these skills at older ages over seven school years, the study also had shortcomings that need to be highlighted. A considerate loss of subjects (54.1%) occurred over the study period, which could have influenced the generalizability of the findings. In order to draw more comprehensive generalizations, similar future studies should be extended to a more representative area as this study was bound to one of nine provinces of SA. School status was also used as a proxy of SES in this study. This had certain limitations and it is recommended that the educational background and income of parents should rather be used as a proxy of SES. Also, the skills score of the CAMSA is not a pure OCS SS score, as it also includes the assessment of locomotor proficiency, which could, therefore, have influenced the score and should also be taken into consideration.

## 5. Conclusions

This study confirmed that competency in object control skills (OCS) should be considered as a key pathway to future physical and motor skill development of children, as it can contribute to a sustained level of capacity to engage in an active lifestyle. Promoting OCS should therefore be considered integral to a holistic view of the development of the child. This demands a focus on creating opportunities to improve competency in a range of OCSs in early childhood development programs. Schools should be made more aware of the value and the role of Physical Education in the prolonged health and development of the child, while the importance of appointing physical education teachers should also be recognized by schools, as their expert knowledge can play an important role in providing sound motor developmental programs to young children. Future longitudinal research is, however, still recommended, which can maybe focus on a longer developmental period as this study mainly focused on the early middle to later childhood period (the primary school years). The consistency or prolonged influences of OCS over the adolescence period, (or during secondary school), which is a known period that influences physical activity levels negatively, especially among girls, should also be studied. Research is also recommended where research designs are drafted in a way that the sustained association and application value of OCS can be measured more accurately in specific sporting settings (e.g., in rugby, soccer, or netball).

## Figures and Tables

**Table 1:** Characteristics of the participants per gender and socio-economic school status (*n* = 374).

	Age		Sex		Socio-Economic School Status			
	*M*	*SD*	*n*	%	Low	%	High	%
	**Boys**							
**Grade 1** **Grade 4** **Grade 7**	6.86 9.91 12.92	0.39 0.41 0.41	178	47.59	90	50.57	88	49.44
	**Girls**							
**Grade 1** **Grade 4** **Grade 7**	6.81 9.86 12.87	0.38 0.42 0.41	196	52.41	124	63.27	72	36.73
	**Total**							
**Grade 1** **Grade 4** **Grade 7**	6.84 9.89 12.90	0.39 0.42 0.41	374	100	214	57.22	160	42.78

Note. *n*—number of total participants; *M*—mean; *SD*—standard deviation; %—percentage; Low—rural environment (low socio-economic status schools); High—urban environment (high socio-economic status schools).

**Table 2:** Descriptive results of OCS SS (Grade 1, 4, and 7) and CAMSA (total score and time in Grade 7) by group and for boys and girls separately.

	Group		Boys		Girls				
	*M*	*SD*	*M*	*SD*	*M*	*SD*	*t*	*p*	Effect Size
**Grade 1**									
OCS SS	7.30	2.31	7.15	2.32	7.43	2.30	1.20	0.230	0.12
**Grade 4**									
OCS SS	9.16	2.38	8.67	2.24	9.62	2.41	3.90	0.001 *	0.39
**Grade 7**									
CAMSA skills total (/14)	10.84	2.00	11.69	1.75	10.06	1.90	−8.60	0.001 *	0.86
CAMSA time (seconds)	16.95	2.46	16.51	2.18	17.36	2.63	3.40 *	0.001 *	0.34

Note. *M*—mean; *SD*—standard deviation; *t*—*t*-value; r ≈ 0.1 small effect, r ≈ 0.3 medium effect, r ≥ 0.5 as a large effect, *p*—*p* value; * *p* < 0.05 = statistical significance.

**Table 3:** Correlations between OCS SS, and the CAMSA skills and time scores (Grade 1, 4, and 7).

Grade 7 CAPL			
Year		Skills Score	Time Score
Grade 1 OCS SS	Boys	0.39 *	−0.29 *
	Girls	0.23 *	−0.18 *
	High SES	0.31 *	−0.27 *
	Low SES	0.13	−0.11
Grade 4 OCS SS	Boys	0.16 *	−0.09
	Girls	0.06	−0.10
	High SES	0.04	−0.16
	Low SES	−0.18	0.05

Note. r ≈ 0.1 * small effect.

**Table 4:** A stepwise regression analysis of grade 1 and grade 4 OCS SS on grade 7 participants CAMSA skills score as adjusted for SES and sex.

*n* = 374	Steps Entered	Beta *	Std. Err. of Beta *	b	Std. Err. of b	*p*-Value	R^2^ -Change
**Grade 1** (R^2^ = 0.326)							
Intercept				9.398 *	0.344 *	0.001 *	
Sex	1	0.383 *	0.043 *	1.533 *	0.173 *	0.001 *	0.166 *
SES	2	−0.296 *	0.044 *	−1.195 *	0.177 *	0.001 *	0.114 *
OCS SS	3	0.220 *	0.044 *	0.191 *	0.038 *	0.001 *	0.046 *
**Grade 4** (R^2^ = 0.281)							
Intercept				10.665 *	0.426 *	0.001 *	
Sex	1	0.371 *	0.046 *	1.483 *	0.182 *	0.001 *	0.166 *
SES	2	−0.335 *	0.045 *	−1.351 *	0.183 *	0.001 *	0.114 *

Note. R^2^-change—coefficient of determination; Beta = standardized regression slope; b—value of the slope; *p*-value—statistical significance; *p* is set at ≤0.05 *.

**Table 5:** A stepwise regression analysis of the variance explained by grade 1 and grade 4 OCS SS on grade 7 participants’ CAMSA time score, controlling for SES and sex.

*n* = 374	Steps Entered	Beta *	Std. Err. of Beta *	B	Std. Err. of b	*p*-Value	R^2^ -Change
**Grade 1** (R^2^ = 0.081)							
Intercept				18.940 *	0.493 *	0.001 *	
OCS SS	1	0.216 *	0.051 *	−0.230 *	0.054 *	0.001 *	0.045 *
Sex	2	0.181 *	0.050 *	−0.892 *	0.248 *	0.001 *	0.035 *
**Grade 4** (R^2^ = 0.043)							
Intercept				17.984 *	0.604 *	0.001 *	
Sex	1	0.181 *	0.053 *	−0.891 *	0.259 *	0.001 *	0.030 *
OCS SS	2	−0.084	0.053	−0.087	0.055	0.111	0.009
SES	3	0.068	0.052	0.337	0.259	0.194	0.004

Note. R^2^-change—coefficient of determination; Beta = standardized regression slope; b * = value of the slope; *p*-value—statistical significance; *p* is set at ≤0.05 *.

## Data Availability

Data available on request due to restrictions. The data presented in this study are available on request from the corresponding author.
